# Evaluation of Changes in Heart Rate Variability Associated with Puppy Growth Using Phase-Rectified Signal Averaging (PRSA) and Non-Gaussianity Index

**DOI:** 10.3390/ani15233449

**Published:** 2025-11-29

**Authors:** Rio Hayashi, Mizuki Hasegawa, Akari Hatanaka, Ahmed S. Mandour, Miki Hirose, Kazumi Shimada, Lina Hamabe, Ken Takahashi, Ryou Tanaka

**Affiliations:** 1 Veterinary Teaching Hospital, Tokyo University of Agriculture and Technology, Tokyo 183-8509, Japan; 2 Department of Animal Medicine (Internal Medicine), Faculty of Veterinary Medicine, Suez Canal University, Ismailia 41522, Egypt; 3 Veterinary Surgery, Department of Veterinary Medicine, Faculty of Agriculture, Tokyo University of Agriculture and Technology, Tokyo 183-8509, Japan; 4 Veterinary Clinical Oncology, Department of Veterinary Medicine, Faculty of Agriculture, Tokyo University of Agriculture and Technology, Tokyo 183-8509, Japan; 5 Department of Pediatrics, Juntendo University Urayasu Hospital, Chiba 279-0021, Japan

**Keywords:** dog, heart rate variability, growth, phase-rectified signal averaging (PRSA), non-Gaussianity of HRV, echocardiogram, ECG, cardiology, veterinary medicine

## Abstract

Heart rate variability (HRV) reflects autonomic nervous system (ANS) activity through nonlinear mechanisms, including complex beat-to-beat fluctuations and chaos-like dynamics of healthy cardiac rhythms. However, conventional HRV indices often show inconsistent interpretations of sympathetic activity across studies, making its evaluation difficult. Recently, nonlinear indices such as Acceleration Capacity (AC), Deceleration Capacity (DC), and the non-Gaussianity index have been introduced to separately evaluate sympathetic and parasympathetic nervous activity. We hypothesized that these indices are applicable in dogs and could reflect age-related changes in ANS function. This study provides the first longitudinal evaluation of nonlinear HRV indices, including AC, DC, and the non-Gaussianity index, during puppy growth, highlighting new insights into autonomic maturation in young dogs. HRV was longitudinally measured in eight healthy puppies (4 to 12 months old) of various breeds every two months. HRV analysis was conducted during sleep using 48-h Holter ECG recordings. We applied frequency-domain (nHF, LF/HF), time-domain (SDNN, RMSSD), geometric (SD1, SD2, SD1/SD2), and nonlinear analyses (AC, DC, non-Gaussianity index). Parasympathetic indicators (RMSSD, SD1) and overall HRV indices (SDNN, SD2) significantly increased with age, while the sympathetic indicator AC significantly decreased. No significant changes were observed in nHF, LF/HF, SD1/SD2, DC, or the non-Gaussianity index. HRV analysis suggests that puppies are initially sympathetic-dominant, shifting toward parasympathetic dominance as they grow. AC may serve as a useful marker of sympathetic activity in young dogs, whereas DC and the non-Gaussianity index may provide stable ANS assessments regardless of age.

## Simple Summary

Heart rate variability (HRV) reflects autonomic nervous system activity, but traditional HRV indices often differ in how they interpret sympathetic function. Recently, new nonlinear indices such as acceleration capacity (AC), deceleration capacity (DC), and the non-Gaussianity index have been proposed to evaluate sympathetic and parasympathetic activity more independently. To examine how these indices change as dogs grow, we monitored HRV in eight healthy puppies from 4 to 12 months of age during sleep using Holter ECG. Parasympathetic indicators such as RMSSD and SD1 increased with age, while AC, a sympathetic indicator, decreased. DC and the non-Gaussianity index showed no clear age-related changes. These findings suggest that autonomic balance shifts from sympathetic to parasympathetic dominance as puppies mature, and that developmental stage should be considered when interpreting HRV in young dogs.

## 1. Introduction

Heart rate variability (HRV) represents fluctuations in R–R intervals and reflects the nonlinear activity of the autonomic nervous system (ANS) [1]. The nonlinear characteristics of HRV arise from complex beat-to-beat fluctuations, chaos-like dynamics, and multiple interdependent regulatory systems operating at different time scales. HRV indices are widely used to assess autonomic function, and reduced HRV is well documented in human cardiovascular diseases, including heart failure, ischemic heart disease, and hypertension [2,3]. In veterinary medicine, HRV has gained attention as well; in dogs, HRV has been reported as a severity marker in myxomatous mitral valve disease and as a potential screening tool for dilated cardiomyopathy in Dobermans and Boxers [4,5]. Disease progression in these conditions induces autonomic imbalance—characterized by sympathetic dominance and reduced vagal tone—which directly contributes to decreased HRV. HRV is influenced by multiple physiological factors such as age, sex, and heart rate (HR) [6,7].

In humans, mean HR declines from birth through adolescence as parasympathetic tone increases and sympathetic activity relatively decreases [8]. A similar age-related decline in HR is observed in dogs, with puppies exhibiting higher HR than adult dogs [9]. Because HR affects HRV both mathematically and physiologically, HR changes during growth are expected to accompany alterations in HRV [6]. However, HRV in dogs under one year of age has not been systematically evaluated, making interpretation in young puppies uncertain. Previous work suggests that puppies exhibit lower parasympathetic tone and higher sympathetic activity compared with adult dogs, gradually shifting toward parasympathetic dominance with maturation [10]. Yet, how this developmental autonomic shift is reflected in HRV remains unclear. Moreover, most evidence arises from cross-sectional studies rather than longitudinal assessments.

The sympathetic and parasympathetic nervous systems interact in a nonlinear and dynamic manner, making it difficult to attribute traditional HRV indices exclusively to one branch of the ANS [11]. This limitation has increased interest in novel HRV analyses capable of differentiating autonomic components. Phase-rectified signal averaging (PRSA) provides acceleration capacity (AC) and deceleration capacity (DC), which correspond to sympathetic and parasympathetic activity, respectively [12]. PRSA-derived indices have demonstrated clinical relevance in human cardiovascular and metabolic diseases and offer advantages such as reduced sensitivity to artifacts [13].

Another emerging metric, the non-Gaussianity index, captures transient HR fluctuations and has been associated with cardiac-related mortality in post-myocardial infarction patients [14]. However, these advanced HRV metrics have not been explored in veterinary populations, and even in human studies, their developmental trajectories remain unclear.

Because HRV during sleep reflects parasympathetic activity more reliably than 24-h recordings in dogs [15], longitudinal sleep HRV measurements may provide more accurate insights into autonomic maturation. Therefore, this study longitudinally evaluated HRV during sleep in healthy puppies under one year of age using traditional HRV indices, PRSA-derived AC and DC, and the non-Gaussianity index to characterize how autonomic maturation is reflected in these metrics.

Therefore, the aim of this study was to longitudinally evaluate autonomic maturation in healthy puppies under one year of age by analyzing HRV during sleep using traditional HRV indices, PRSA-derived AC and DC, and the non-Gaussianity index. To the best of our knowledge, this is the first study to longitudinally assess these novel HRV metrics in growing dogs, providing new insights into how autonomic maturation is reflected in both linear and nonlinear HRV parameters.

## 2. Materials and Methods

### 2.1. Experimental Animals

**Figure 1:**
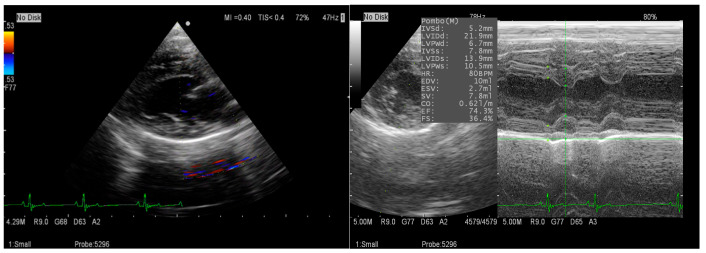
Representative echocardiographic image used for health screening, showing no detectable cardiac abnormalities in the examined dog.

This study was conducted at Tokyo University of Agriculture and Technology. Eight healthy client-owned dogs (two males, six females; Toy Poodle, Miniature Dachshund, Miniature Schnauzer, Maltese, Chihuahua, French Bulldog) were examined at 4, 6, 8, 10, and 12 months of age. All dogs had normal physical and echocardiographic findings (Figure 1). Dogs were housed individually with free access to water and fed twice daily. HRV recordings were obtained during sleep on the second day after Holter attachment. Body weight (mean ± SD) at each age was 2.0 ± 1.0 kg (4 months), 3.3 ± 1.5 kg (6 months), 4.1 ± 2.1 kg (8 months), 4.3 ± 2.3 kg (10 months), and 4.4 ± 2.4 kg (12 months). Written informed consent was obtained from all owners, and all procedures were non-invasive.

### 2.2. Placement and Removal of the Holter ECG Device

**Figure 2:**
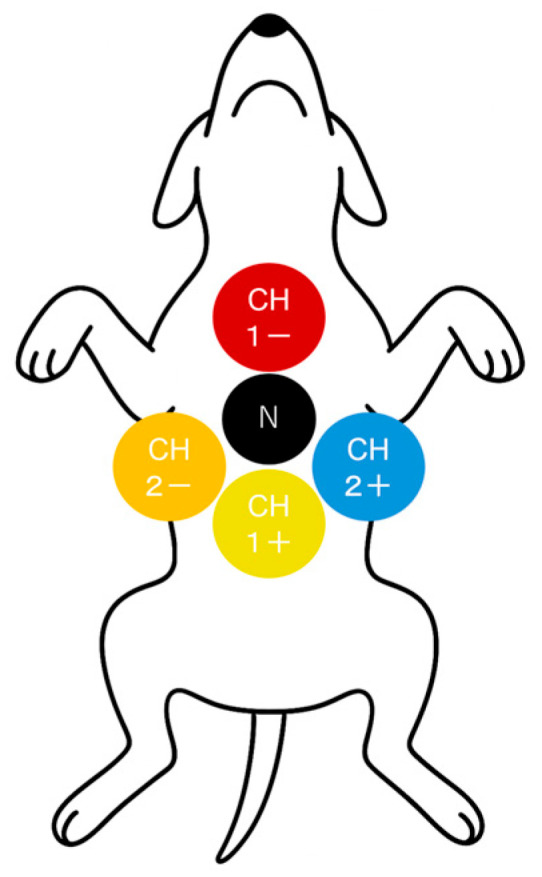
Electrode placement for ECG recording. Red: manubrium; ground: midline; yellow: xiphoid; blue: left 5th–6th intercostal space; orange: right 5th–6th intercostal space.

Dogs were placed in dorsal recumbency, and the hair over the sternum and bilateral 5th–6th intercostal spaces was clipped to allow electrode attachment. After skin preparation, five ECG electrode pads were positioned over the manubrium sterni, xiphoid process, the midpoint between them, and the left and right 5th–6th intercostal spaces. Electrodes were secured using an adhesive elastic bandage, and the Holter ECG recorder (Nihon KohdenRAC-5203, Tokyo, Japan) was fixed on the dog’s back with additional bandaging. A protective garment was worn to prevent displacement of the device (Figure 2).

The dogs were then moved to a cage, and Holter ECG recordings were obtained continuously for 48 h. After completion of recording, the protective garment and bandages were carefully removed, followed by gentle removal of the electrode pads to minimize skin irritation.

### 2.3. ECG Data Processing

**Figure 3:**
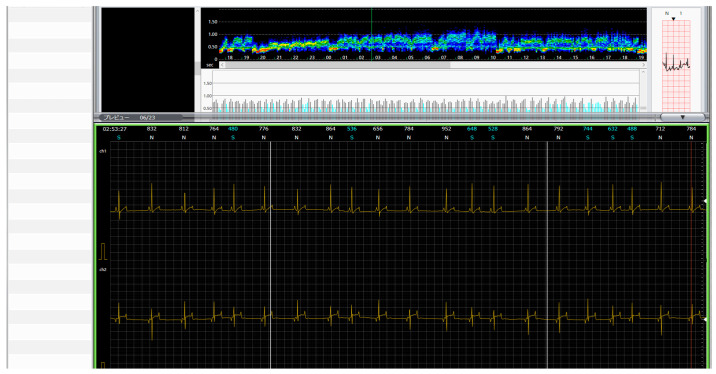
Representative Holter ECG waveform showing normal sinus rhythm with clear R-wave identification.

Holter data were analyzed using Nihon Kohden long-term ECG analysis software. R-wave detection was automatically performed and manually corrected when necessary. A representative raw ECG waveform is shown in Figure 3.

### 2.4. Standard HRV Analysis

HRV analysis was conducted using MATLAB (MathWorks, R2022a, United States) based on algorithms from Juntendo University. Data from 00:00 to 08:00 on the second day were analyzed as sleep phase data. Mean heart rate (mean HR) was obtained from the Holter ECG recordings. Linear analysis included both time-domain and frequency-domain analyses [11]. In time-domain analysis, the standard deviation of NN intervals over 24 h (SDNN, ms) and the root mean square of successive differences between adjacent NN intervals (RMSSD, ms) were calculated. In frequency-domain analysis using fast Fourier transform, the low-frequency (LF) and high-frequency (HF) components were obtained, and normalization was performed to reduce within- and between-subject variability and enhance reproducibility. Normalized high-frequency power (nHF, ms^2^) and normalized low-frequency power (nLF ms^2^) were calculated using the equations nHF = HF/(LF + HF) and nLF = LF/(LF + HF) [16].

For nonlinear analysis, geometric analysis, non-Gaussianity index, and phase-rectified signal averaging (PRSA) were performed. Geometric analysis included Poincaré plot analysis [17,18]. A Poincaré plot is graphed by plotting every R-R interval against the prior interval, creating a scatter plot. The standard deviation (hence SD) of the distance of each point from the y = x axis (SD1, ms), specifies the ellipse’s width. The standard deviation of each point from the y = x + average R–R interval (SD2, ms) specifies the ellipse’s length. The ratio of these parameters (SD1/SD2) was also calculated.

### 2.5. HRV Analysis Using Novel Methods

HRV analysis was conducted using MATLAB based on Juntendo University algorithms. Data from 00:00 to 08:00 on the second day were analyzed as sleep phase data. PRSA is used to detect subtle short-term repetitive patterns in time-series signals typically obscured by non-stationarity, noise, and artifacts. In ECG analysis, R-R intervals shorter than the preceding R-R interval were identified as acceleration anchors, while those longer were identified as deceleration anchors. R-R intervals with over 20% deviation from the preceding RR interval were excluded from anchoring to remove artifacts. The PRSA segments were aligned based on anchors, and the segments were averaged. Acceleration capacity (AC) and deceleration capacity (DC) were calculated using the formula [19]:

AC or DC = {(RR(0) + RR(1) − RR(−1) − RR(−2))}/4

where RR(0) is the anchored R-R interval, RR(1) is the subsequent R-R interval, and RR(−1) and RR(−2) are the preceding R-R intervals.

The non-Gaussianity index quantifies the non-Gaussian behavior of HRV, characterized by pronounced fat tails and peak distributions. HRV intermittency is explained by increased probability of both large and small increments compared to Gaussian fluctuations. The background and mathematical framework of the non-Gaussianity index have been established [20,21], and analysis was conducted accordingly.

### 2.6. Statistical Analysis

This statistical approach was conducted with reference to the methods described in reference [22]. All data were expressed as median (interquartile range, IQR). Friedman’s test was used to compare HRV indices across different ages (4, 6, 8, 10, and 12 months), followed by Dunn’s test for multiple comparisons. Statistical significance was set at *p* < 0.05. Statistical analyses and graphical representations were performed using GraphPad Prism 8 (GraphPad Software, San Diego, CA, USA).

## 3. Results

All dogs remained alive and showed no clinical abnormalities throughout the study period.

The detailed median (IQR) values for all HRV parameters at each age are summarized in Table 1.

### 3.1. Changes in Mean Heart Rate (meanHR)

**Figure 4:**
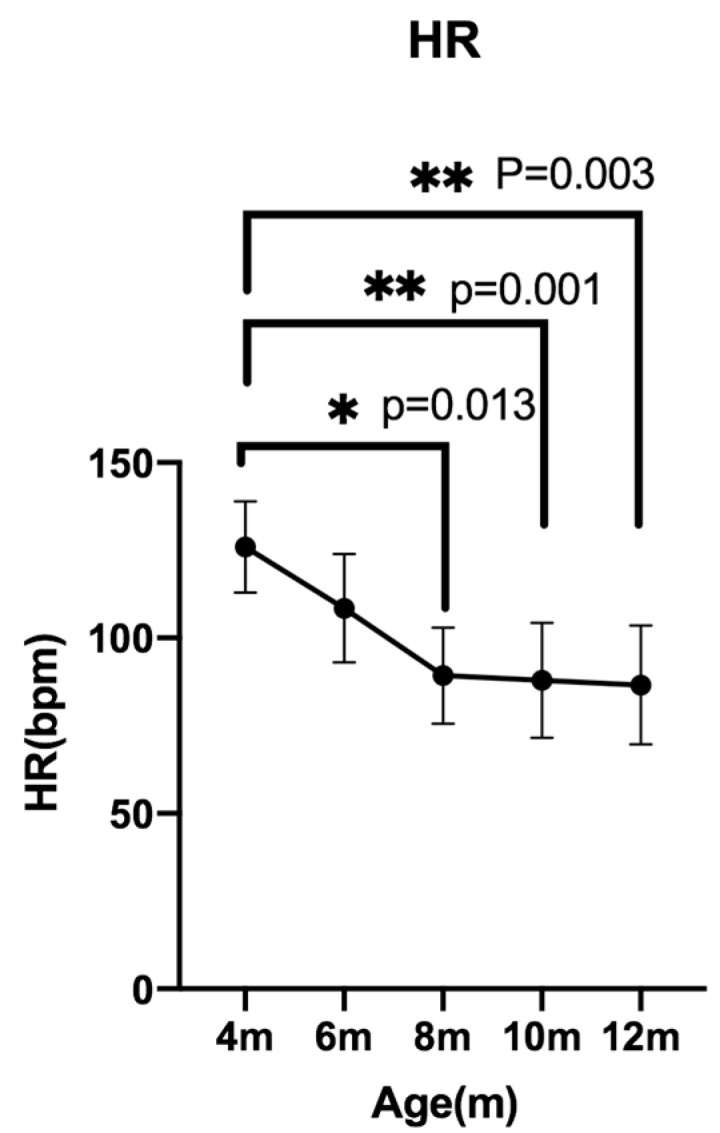
Changes in mean heart rate measured bimonthly from 4 to 12 months of age using Holter electrocardiography. Data are presented as median ± IQR.

A significant overall difference was detected using the Friedman test (*p* < 0.05). Subsequent pairwise comparisons revealed significant differences between 4 and 8 months (*p* = 0.0132), 4 and 10 months (*p* = 0.0010), and 4 and 12 months (*p* = 0.0039) (Figure 4).

### 3.2. Time-Domain Analysis

The **SDNN** values were 95.58 (66.33–120.1) at 4 months, 131.6 (76.66–187.8) at 6 months, 196.6 (149.4–213.8) at 8 months, 219.0 (175.5–245.2) at 10 months, and 213.0 (181.1–293.9) at 12 months. The Friedman test indicated a significant difference (*p* < 0.05). Pairwise comparisons showed significant differences between 4 and 10 months (*p* = 0.0090), 4 and 12 months (*p* = 0.0028), 6 and 10 months (*p* = 0.0157), and 6 and 12 months (*p* = 0.0050).

**Figure 5:**
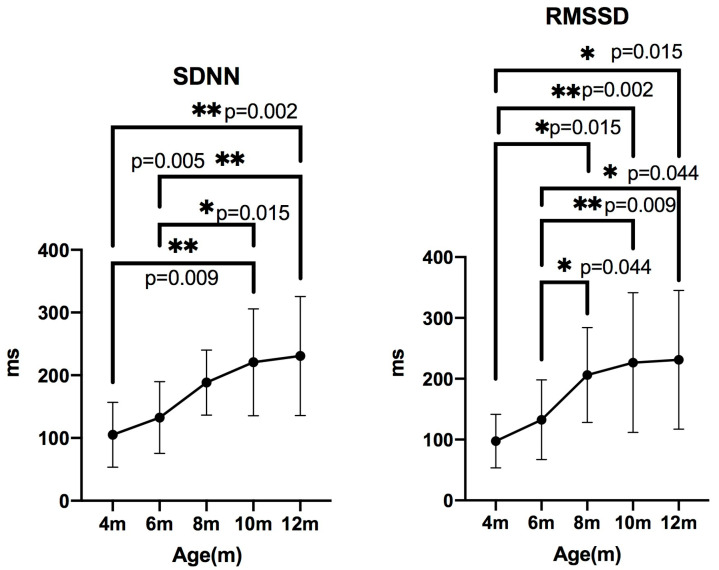
Changes in time-domain indices measured every two months by Holter electrocardiography, presented as median ± IQR. SDNN: standard deviation of NN intervals; RMSSD: root mean square of successive differences.

For **RMSSD**, values were 97.97 (56.30–115.9) at 4 months, 128.1 (72.52–177.3) at 6 months, 202.0 (144.5–254.9) at 8 months, 198.4 (162.4–273.3) at 10 months, and 208.4 (166.5–283.4) at 12 months. A significant difference was also observed by the Friedman test (*p* < 0.05). Pairwise comparisons revealed significant differences between 4 and 8 months (*p* = 0.0157), 4 and 10 months (*p* = 0.0028), 4 and 12 months (*p* = 0.0157), 6 and 8 months (*p* = 0.0443), 6 and 10 months (*p* = 0.0090), and 6 and 12 months (*p* = 0.0443) (Figure 5).

### 3.3. Frequency-Domain Analysis

The **nHF** values were 0.2228 (0.1466–0.2758) at 4 months, 0.2558 (0.1726–0.2773) at 6 months, 0.2768 (0.2236–0.3107) at 8 months, 0.2998 (0.2557–0.3369) at 10 months, and 0.3050 (0.2562–0.3258) at 12 months.

The **LF/HF** ratios were 0.7310 (0.4682–1.227) at 4 months, 0.6536 (0.5043–0.9927) at 6 months, 0.4740 (0.3923–0.8336) at 8 months, 0.4468 (0.3808–0.5726) at 10 months, and 0.4695 (0.3192–0.8455) at 12 months.

**Figure 6:**
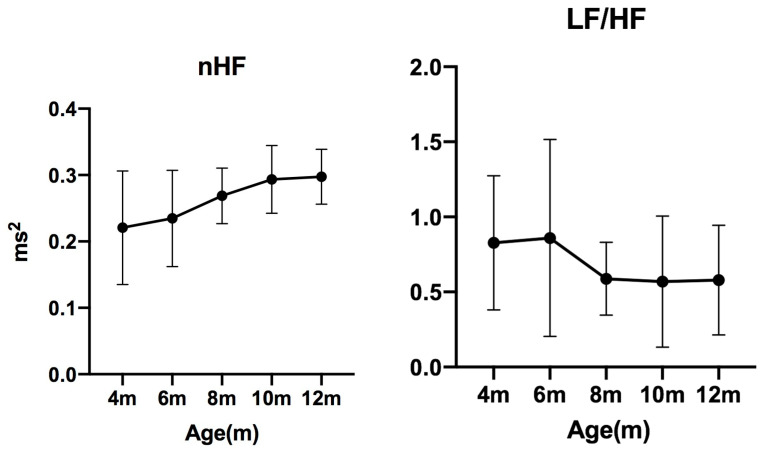
**Changes in frequency-domain indices measured every two months (median ± IQR).** nHF: normalized high-frequency power (0.15–0.4 Hz); LF/HF: low- to high-frequency power ratio.

No significant differences were observed in either **nHF** or **LF/HF** (Figure 6).

### 3.4. Nonlinear Analysis

#### 3.4.1. Geometric Analysis

The **SD1** values were 69.34 (39.84–82.00) at 4 months, 90.70 (51.33–125.5) at 6 months, 143.0 (102.3–180.5) at 8 months, 140.4 (115.0–193.5) at 10 months, and 147.5 (117.9–200.7) at 12 months. The Friedman test showed a significant difference (*p* < 0.05). Pairwise comparisons revealed significant differences between 4 and 8 months (*p* = 0.0157), 4 and 10 months (*p* = 0.0028), 4 and 12 months (*p* = 0.0157), 6 and 8 months (*p* = 0.0443), 6 and 10 months (*p* = 0.0090), and 6 and 12 months (*p* = 0.0443).

The **SD2** values were 115.1 (85.15–148.0) at 4 months, 157.0 (97.89–235.8) at 6 months, 229.9 (175.0–269.7) at 8 months, 262.8 (219.1–307.8) at 10 months, and 259.6 (221.8–363.7) at 12 months. A significant difference was also detected (*p* < 0.05), with pairwise differences between 4 and 10 months (*p* = 0.0090), 4 and 12 months (*p* = 0.0028), 6 and 10 months (*p* = 0.0157), and 6 and 12 months (*p* = 0.0050).

**Figure 7:**
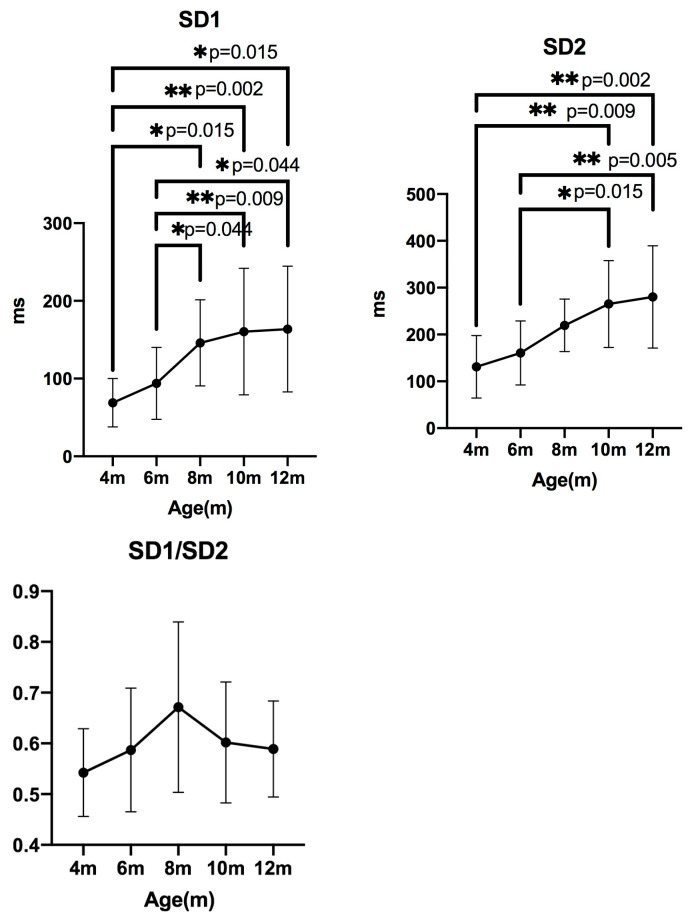
**Changes in geometrical analysis indices measured every two months (median ± IQR).** SD1: short-term HRV; SD2: long-term HRV; SD1/SD2: ratio of short- to long-term variability.

The **SD1/SD2** ratios showed no significant changes (Figure 7).

#### 3.4.2. PRSA and Non-Gaussianity Index Analysis

The **AC** values were −8.327 (−10.33 to −7.404) at 4 months, −10.86 (−11.72 to −7.296) at 6 months, −12.48 (−13.88 to −10.94) at 8 months, −12.36 (−13.27 to −12.17) at 10 months, and −13.12 (−14.47 to −10.90) at 12 months.

A significant difference was detected (*p* < 0.05), with pairwise comparisons revealing a significant difference between 4 and 8 months (*p* = 0.0406).

The **DC** values were 7.807 (7.565–8.894) at 4 months, 9.610 (6.873–10.59) at 6 months, 12.11 (10.27–13.50) at 8 months, 11.47 (10.97–14.58) at 10 months, and 13.34 (9.032–14.76) at 12 months.

**Figure 8:**
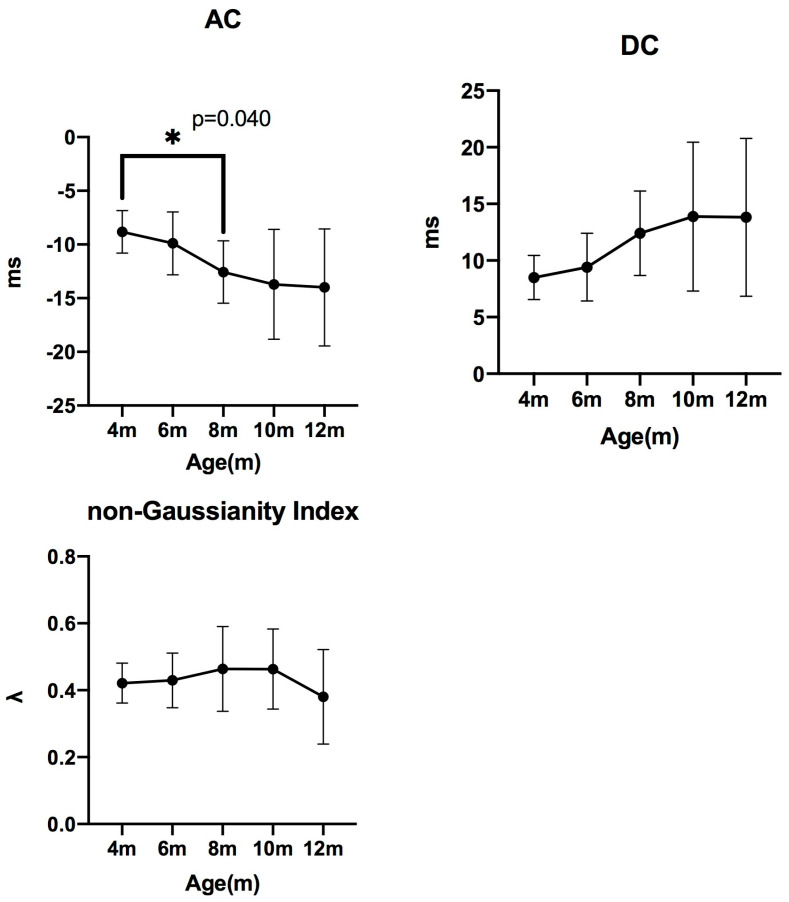
**Changes in PRSA-derived indices and non-Gaussianity index measured every two months (median ± IQR).** AC: acceleration capacity; DC: deceleration capacity; non-Gaussianity index: non-Gaussianity of HRV.

The **non-Gaussianity index** values were 0.4146 (0.3643–0.4830) at 4 months, 0.4416 (0.3481–0.4675) at 6 months, 0.4486 (0.4010–0.5219) at 8 months, 0.4461 (0.3656–0.5831) at 10 months, and 0.3794 (0.2865–0.4252) at 12 months (Figure 8).

No significant changes were observed in DC or the non-Gaussianity index.

## 4. Discussion

Heart rate variability (HRV) represents autonomic modulation, and previous human and canine studies have shown that HRV is influenced by nonlinear autonomic changes and exhibits spatial and temporal complexity under physiological conditions [1]. Puppies are known to have higher heart rates than adult dogs [9], and the present study confirmed an age-related decline in heart rate, consistent with earlier findings. Because HRV is dependent on heart rate [6], the developmental HRV changes observed in puppies align with physiological expectations.

Conventional HRV indices such as SDNN reflect overall autonomic activity and are widely used in humans, where SDNN > 100 ms is considered normal [11]. However, canine reference values are not well established, with only a few reports describing mean SDNN values of 287.80 ms [23] and 155 ms [24] in healthy adult dogs. In contrast, SDNN values in 4- and 6-month-old puppies in this study were considerably lower, suggesting immature autonomic regulation during early growth. Similar age-related increases in SDNN have been described in human children [8], although the developmental timeline differs between species. Thus, SDNN interpretation in young dogs must consider age.

Parasympathetic-related indices such as RMSSD and its nonlinear analogue SD1 [25], which correlate with high-frequency (HF) power [26], also increased with age in this study. However, nHF did not show significant differences among age groups. This divergence between RMSSD/SD1 and nHF is consistent with previous reports showing that HF power is strongly affected by respiratory sinus arrhythmia (RSA), which is more pronounced in dogs than in humans [27,28,29]. Moreover, RMSSD is reported to be less influenced by RSA compared with other parasympathetic indices [30], suggesting that RMSSD may more accurately reflect developmental changes in vagal tone than nHF.

Phase-rectified signal averaging (PRSA) provides indices that separately quantify sympathetic (AC) and parasympathetic (DC) activity in humans. Human data indicate that AC and DC values in neonates aged 1–4 days are −3.58 ± 0.67 and 3.38 ± 0.57 [31], respectively, and that these indices further change in children aged 4–17 years (AC = −9.5 ± 1.09; DC = 8.3 ± 0.93). These findings suggest that AC and DC are influenced by developmental stage. Consistent with this concept, the present study demonstrated that AC decreased until 8 months of age in puppies, implying that AC may also undergo age-related modulation in dogs. DC did not show significant age-related changes, unlike in human pediatric data. These species-specific differences indicate that PRSA indices may reflect different developmental mechanisms in dogs and emphasize the need for canine-specific reference ranges. Although the physiological interpretation of AC and DC in dogs has not been fully established, previous human studies and the present findings suggest that these indices may reflect similar autonomic regulatory mechanisms. Nevertheless, the clear age-related decline in AC highlights its potential value as a sympathetic marker in veterinary practice. Although the age-related decrease in AC is physiologically expected as part of autonomic maturation, our study is the first to longitudinally characterize these developmental trajectories using PRSA-derived indices in growing dogs. This provides novel quantitative insight into sympathetic maturation that has not been previously documented in canine studies.

The non-Gaussianity index, a nonlinear HRV measure associated with increased mortality after acute myocardial infarction in humans (0.40 in survivors vs. 0.80 in cardiac deaths) [14], did not vary significantly across age groups in our study and remained lower than values reported in patients who died from cardiac causes [14,32]. Although this index may reflect sympathetic overactivation, the lack of developmental change in puppies suggests limited sensitivity to normal autonomic maturation. Therefore, while the non-Gaussianity index reflects excessive sympathetic activation, it did not show significant age-related changes during growth.

Overall, the present results demonstrate that autonomic maturation occurs dynamically during the first year of life in dogs. Several indices—including SDNN, RMSSD, SD1, and AC—captured age-related changes, indicating that these parameters should be interpreted with consideration of developmental stage.

This study has limitations. Respiratory rate was not measured during Holter recordings, limiting adjustment of the HF range and PRSA interpretation. Because PRSA indices may be sensitive to respiratory influences [33], simultaneous respiratory monitoring would improve accuracy. Nevertheless, previous reports indicate that resting respiratory rates during sleep are similar in healthy dogs (mean 13 breaths/min, max 23 breaths/min) [34] and humans (11–21 breaths/min) [35], suggesting minimal species differences. Therefore, although respiratory rate was not recorded, its impact on the present HRV results is likely limited. Future studies incorporating respiratory measurement and larger sample sizes will further clarify developmental trajectories of HRV indices and help establish robust age-related reference values in dogs.

## 5. Conclusions

In this study, we characterized developmental changes in HRV during the first year of life in dogs using conventional indices, PRSA-derived parameters, and a nonlinear measure. We confirmed that HRV increases with maturation and demonstrated, for the first time, age-related changes in AC in growing dogs. These findings indicate that AC is a measurable PRSA-derived index in the canine species and may support the assessment of sympathetic modulation in future research.

When HRV is evaluated in puppies under one year of age, age should be carefully considered, as autonomic maturation is ongoing during this period. Although further validation in clinical populations is required, the present results provide a foundation for exploring the potential utility of PRSA indices, including AC, in cardiovascular conditions such as MMVD or DCM. Overall, our findings contribute to establishing age-aware HRV interpretation in veterinary medicine and offer a basis for future clinical applications.

## 6. Limitations

This study has several limitations. Although multiple breeds were included, none of the breeds known to show breed-specific HRV characteristics were present. All dogs were small-breed dogs, and previous studies indicate that sex, body size, and brachycephalic status have minimal effects on HRV. Therefore, these factors were unlikely to have influenced the present findings. The lack of respiratory rate measurement is a limitation, especially for interpreting HF and LF/HF and PRSA-based indices. However, reported respiratory rates in healthy sleeping dogs provide a reasonable reference, and all dogs were small breeds without known breed-related respiratory differences, suggesting minimal impact on our findings.

## Figures and Tables

**Table 1:** Detailed summary of HRV measurements across all ages. Median (IQR) values for all heart rate variability parameters obtained every two months from 4 to 12 months of age. The table contains time-domain indices (SDNN, RMSSD), frequency-domain indices (nHF, LF/HF), geometric indices (SD1, SD2, SD1/SD2), PRSA-derived indices (AC, DC), and the non-Gaussianity index.

Parameter Category	Indicators Median (IQR)	4 m	6 m	8 m	10 m	12 m
	Mean HR (bpm)	127.5 (116.0–135.3)	114.4 (94.03–122.3)	90.97 (72.69–99.50)	87.98 (85.59–97.76)	90.91 (80.53–95.85)
Time-Domain	SDNN (ms)	95.58 (66.33–120.1)	131.6 (76.66–187.8)	196.6 (149.4–213.8)	219.0 (175.5–245.2)	213.0 (181.1–293.9)
	RMSSD (ms)	97.97 (56.30–115.9)	128.1 (72.52–177.3)	202.0 (144.5–254.9)	198.4 (162.4–273.3)	208.4 (166.5–283.4)
Frequency- Domain	nHF (ms^2^)	0.2228 (0.1466–0.2758)	0.2558 (0.1726–0.2773)	0.2768 (0.2236–0.3107)	0.2998 (0.2557–0.3369)	0.3050 (0.2562–0.3258)
	LF/HF	0.7310 (0.4682–1.227)	0.6536 (0.5043–0.9927)	0.4740 (0.3923–0.8336)	0.4468 (0.3808–0.5726)	0.4695 (0.3192–0.8455)
Geometric	SD1 (ms)	69.34 (39.84–82.00)	90.70 (51.33–125.5)	143.0 (102.3–180.5)	140.4 (115.0–193.5)	147.5 (117.9–200.7)
	SD2 (ms)	115.1 (85.15–148.0)	157.0 (97.89–235.8)	229.9 (175.0–269.7)	262.8 (219.1–307.8)	259.6 (221.8–363.7)
	SD1/SD2	0.5478 (0.4752–05679)	0.5691 (0.4801–0.6664)	0.6167 (0.5588–0.8584)	0.5996 (0.5213–0.7093)	0.5986 (0.4912–0.6610)
PRSA	AC (ms)	−8.327 (−10.33–−7.404)	−10.86 (−11.72–−7.296)	−12.48 (−13.88–−10.94)	−12.36 (−13.27–−12.17)	−13.12 (−14.47–−10.90)
	DC (ms)	7.807 (7.565–8.894)	9.610 (6.873–10.59)	12.11 (10.27–13.50)	11.47 (10.97–14.58)	13.34 (9.032–14.76)
Non-Gaussianity	Non-Gaussianity Index (λ)	0.4146 (0.3643–0.4830)	0.4416 (0.3481–0.4675)	0.4486 (0.4010–0.5219)	0.4461 (0.3656–0.5831)	0.3794 (0.2865–0.4252)

## Data Availability

The original contributions presented in this study are included in the article. Further inquiries can be directed to the corresponding author.

## Abbreviations

HRV	Heart rate variability
HR	Heart rate
ANS	Autonomic nervous system
RSA	Respiratory sinus arrhythmia
PRSA	Phase rectified signal averaging
SD1	The distance of each point from the y = x axis
SD2	The standard deviation of each point from the y = x + average R–R interval
SDNN	The standard deviation of NN intervals
RMSSD	The root mean square of successive differences between adjacent NN intervals
DC	Deceleration capacity
AC	Acceleration capacity
nHF	Normalized high-frequency power
LF/HF	Lower-frequency/high-frequency power ratio
ECG	Electrocardiogram
IQR	Interquartile range
SD	Standard deviation
